# Influence of alpine mountain climate of Bavaria on patients with atopic diseases: studies at the Environmental Research Station Schneefernerhaus (UFS - Zugspitze) – a pilot study

**DOI:** 10.1186/2045-7022-4-17

**Published:** 2014-05-08

**Authors:** Bernadette Eberlein, Johannes Huss-Marp, Florian Pfab, Rainald Fischer, Regina Franz, Michele Schlich, Maria Leibl, Veronika Allertseder, Jarmila Liptak, Marie Kriegisch, Romain Hennico, Julia Latotski, Cordula Ebner von Eschenbach, Ulf Darsow, Jeroen Buters, Heidrun Behrendt, Rudolf Huber, Johannes Ring

**Affiliations:** 1 Department of Dermatology and Allergy Biederstein, Technical University Munich, Munich, Germany; 2 Center of Allergy and Environment (ZAUM, Zentrum Allergie und Umwelt), Technische Universität and Helmholtz Center, Munich, Germany; 3 Division of Respiratory Medicine Campus Innenstadt, Ludwig-Maximilians-Universität München, Munich, Germany

**Keywords:** Moderate-altitude mountain climate, Zugspitze, Rhinoconjunctivitis, Atopic eczema, Asthma, Environmental Research Station Schneefernerhaus, UFS

## Abstract

Mountain and maritime climate therapy takes advantage of specific climatic conditions to treat chronic allergic diseases. It was the aim of the study to investigate effects of a 5 day sojourn on atopic diseases at the highest German mountain. In this pilot study 18 patients with grass pollen-induced rhinoconjunctivitis, atopic ezcema or asthma and 11 non-allergic controls were included. Skin physiology parameters, changes of the respiratory and nasal functions, subjective symptoms and blood parameters were measured during a 5-day observation period in the Environmental Research Station Schneefernerhaus (UFS) at the moderate altitude mountain region (Zugspitze; 2650 m alt.) compared to a low altitude area (Munich; 519 m alt.). Several of the skin physiology parameters changed significantly during the observation period (decrease of skin hydration, increase of skin smoothness, skin roughness, skin scaliness and pH-value). In patients with atopic eczema, the SCORAD (Severity Scoring of Atopic Dermatitis) and the scores of the DIELH (Deutsches Instrument zur Erfassung der Lebensqualität bei Hauterkrankungen) did not change significantly. Histamine induced itch decreased significantly. Parameters of nasal function did not change significantly. Several lung parameters showed a slight, but statistically significant improvement (forced expiratory volume in one second/volume capacity [FEV1/VC], peak expiratory flow [PEF], maximum expiratory flow at 50% of vital capacity [MEF 50], maximal mid-expiratory flow between 25% and 75% of vital capacity [MMFEF 25/75]), whereas the vital capacity (VC) decreased significantly. ECP (eosinophil cationic protein) in the serum and parameters of blood count changed significantly. These results show that the benefit of a moderate altitude mountain climate sojourn over a period of 5 days differs in depending on the atopic disease. Especially asthma parameters and itching of the skin improved. It would be interesting to assess the parameters during longer observation periods in alpine climate.

## Findings

Climate therapy comprises the use of certain climatic conditions in the treatment of chronic diseases. In allergy, maritime and moderate altitude mountain zones are of interest [1,2]. It was the aim of this pilot study to follow objective and subjective parameters in patients suffering from rhinoconjunctivitis and/or atopic eczema and asthma over a 5 days sojourn in the Environmental Research Station Schneefernerhaus (UFS) at the Zugspitze (2650 m alt.) in the alpine mountain climate of Bavaria compared to lowland in Munich (518 m alt.).

18 patients (6 males, 12 females; mean age: 26 years, range: 18–32 years) with grass-pollen induced rhinoconjunctivitis, atopic eczema or asthma and 11 non-allergic controls (4 males, 7 females; mean age: 30 years, range 24–43 years) were included in the study. Characteristics of patients are shown in Table 1.

**Table 1:** Clinical characteristics, skin tests, determination of total and specific lgE in the patient group

**Patient number**	**Age (years)**	**Sex**	**Atopic disease**	**Prick-test (positive)**	**Total IgE (IU/ml)**	**Specific IgE (CAP-class)**
01	31	f	Asthma, AR	Birch, grass, cat, celery	275	D. pter. 2, cat 2, dog 3, hazelnut 2, celery 1, grass 4, birch 4, hazel 3
02	25	f	AR	D. pter., birch, grass, mugwort	964	D. pter. 3, celery 3, grass 6, birch 4, hazel 5, wheat flour 3, rye 5, mugwort 3
03	25	f	AR	D. pter., cat, grass, mugwort	79.4	D. pter. 3, cat 1, grass 2, rye 2, mugwort 2
04	31	f	AR	Hazel, celery, Alt. alternata	14.2	Grass 2
05	23	f	Asthma, AR	D. pter., birch, dog, cat	134	D. pter. 4, cat 2, dog 2, grass 3
06	24	f	AE, Asthma, AR	Grass	161	D. pter. 5, grass 2, mugwort 1, Ambrosia 1
07	24	f	AE, Asthma	Trees, mugwort, birch, D. pter., alder, grass, hazel	2248	D. pter. 4, cat 3, celery 3, grass 3, birch 5, wheat flour 3, mugwort 3, Ambrosia 3, latex 2
08	30	m	AE, AR	Trees, birch, D. pter., alder, grass, hazel, dog, cat	1822	D. pter. 6, cat 4, grass 5, birch 3, wheat flour 1, mugwort 1, milk 2
09	28	m	AR	Trees, mugwort, birch, Blatella, D. far., D. pter., alder, grass, hazel, dog, cat, ambrosia, herbage	226	D. pter. 4, cat 2, hazel nut 3, grass 2, birch 3, mugwort 2, Ambrosia 1
10	25	f	AD	Grass, cat, herbage	113	Cat 3, grass 4
11	18	m	Asthma	D. far., D. pter., grass	493	D. pter. 2, grass 5, Ambrosia 1
12	20	m	AE, Asthma, AR	Trees, birch, D. far., D. pter., alder, hazel, Aspergillus, Ambrosia	56.2	D. pter., 3, birch 3
13	20	f	AE, Asthma	Negative	136	Negative
14	27	m	AE, Asthma, AR	Trees, birch, D. pter., alder, grass, hazel, dog, cat, horse, Alternaria tenuis, Ambrosia	1455	D. pter. 3, cat 5, grass 3, birch 6, wheat flour 2, mugwort 3, egg white 2, codfish 2, Cladosporium herbarum 3
15	24	f	AE, Asthma, AR	Trees, birch, D. far., D. pter., alder, grass, hazel, dog, cat, herbage, horse	159	D. pter. 4, cat 2, grass 2, birch 3
16	26	f	AD	Mugwort, grass, herbage	8.7	Grass 3
17	32	m	AR	Trees, birch, alder, grass, herbage, Ambrosia	142	D. pter. 1, cat 2, grass 4, birch 2, wheat flour 1, Ambrosia 2
18	28	f	Asthma, AR	Trees, birch, D. far., D. pter., alder, grass, hazel, herbage,	77.5	D. pter. 3, celery 1, grass 3, birch 4, Ambrosia 2

*Abbreviations*: *AD* atopic diathesis, *AE* atopic eczema, *AR* allergic rhinitis, *D. far.* Dermatophagoides farinae, *D. pter.* Dermatophagoides pteronyssinus, *f* female, *m* male.

Five-day observation periods at the Environmental Research Station Schneefernerhaus (UFS) were performed at the following time points with groups up to 10 patients and/or controls: July/August 2008, March 2009 and July 2009. All parameters were measured 3 to 4 days in Munich before the sojourn at the UFS (t1), at the first and second day (t2) and at the fourth and fifth (t3) day during the sojourn at the UFS as well as about 4 weeks later in Munich (t4). The local Ethical Committee approved the study (Ethik-Kommission der Bayerischen Landesärztekammer, No. 08054). All participants had given informed consent. All parameters were measured in all patients (exception: SCORAD only in patients with atopic eczema) and controls (exceptions: skin prick test titration, SCORAD, conjunctival provocation).

The severity of the atopic eczema was graded according to the SCORAD [3]. A skin prick test titration with different concentrations of the grass pollen extract was performed in patients at all time points and the wheal and flare reactions were measured. Itch intensity after prick testing of histamine was rated on a computerized visual analogue scale (VAS).

Parameters of skin physiology (surface pH, sebum, skin hydration) were measured on the flexor side of the forearm with a Corneometer (CORNEOMETER CM825/SEBUMETER SM 810/SKIN-pH-METER PH 900 KOMBI, Courage and Khazaka electronic GmbH, Köln, Germany).

Transepidermal water loss (Tewameter TM 300; CK electronic GmbH, Köln, Germany) was measured in unexposed skin and 10 min after exposure to a 0.5 M NaOH solution (alkali resistance test) and 0.9% NaCl solution 2 times for 10 min with an interval of 10 min in between. Laser Doppler imaging (Moor Instruments, Axminster, England) was applied to monitor dermal blood flow.

Application of skin replicas was done according to previous publications [4]. Replicas were measured by means of the optical skin measuring system VisioScan VC98 with the software SELS 2000 (Courage and Khazaka electronic GmbH, Köln, Germany) calculating skin surface parameters.

In all subjects rhinomanometric and pulmonary function parameters were determined with a spirometer (Flowscreen Pro, Jaeger GmbH, Hoechberg, Germany) and a methacholine challenge test was performed with a bodyplethysmograph (Master Screen Body, Jaeger GmbH, VIASYS Healthcare, Hoechberg, Germany) as previously described [5]. Peak-flow monitoring was done with peak-flow-meters (Mini-Wright-Peak-Flow-Meter, Clemente Clark, Essex, England). FE_NO_ measurements (NIOX MINO; Aerocrine, Solna, Sweden) were performed at the different time points as previously described [6].

Nasal secretions were collected by placing small cotton wool pieces into the middle meatus ot the nose for 20 minutes, followed by centrifugation (3000 R, 20 min) of the cotton wool pieces. In all samples ECP was analyzed (CAP ECP FEIA, Pharmacia, Uppsala, Sweden).

In the conjunctival provocation test five serial dilutions (1:10) of grass pollen extracts were created. A single drop (20 μl) of the lowest concentration was placed in the conjunctival sac followed by the next concentration at 10-min intervals switching from one eye to the other until symptoms appeared. Symptoms were scored as absent (0,) mild (1), moderate (2) or severe (3) 5 and 10 min after challenge.

The following questionnaires were used: Deutsches Instrument zur Erfassung der Lebensqualität bei Hauterkrankungen (DIELH), a questionnaire for health related quality of life (SF-36), The Eppendorf Itch Questionnaire (EIQ) in adults) and the Rhinoconjunctivitis Quality of Life Questionnaire (RQLQ) [7-10].

The following blood parameters were determined: complete blood count (Sysmex XT-2000 i/XT-1800i; Sysmex Corporation, Japan), eosinophil cationic protein (CAP ECP FEIA, Pharmacia, Uppsala, Sweden), human IL-16 (DuoSet ELISA Development System, R&D Systems Europe, Abingdon, United Kingdom) and IL-33 (Human IL-33 ELISA Quantitation Kit, Gentaur, Brussels, Belgium).

Data were analyzed using SPSS. For statistical analysis of the whole study population the Friedman’s one-way analysis of variance by ranks was used for paired samples and the Wilcoxon test for unpaired samples. For the disease-related analysis the univariate analysis of variance (ANOVA) was used. The critical value for significance was set at P < 0.05 for all analyses.

**Figure 1:**
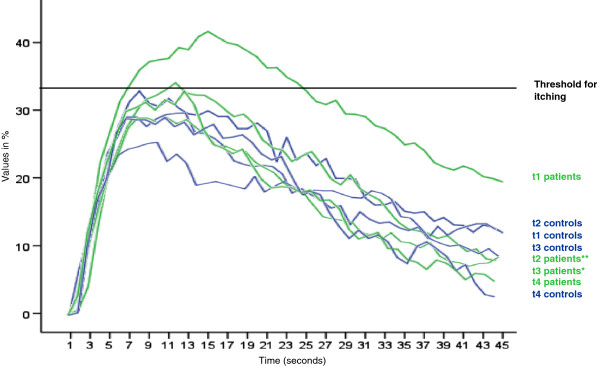
**Mean of itch intensity after skin prick test with histamine of patients and controls at the different time points (Munich: time point 1 (t1) and time point 4 (t4); UFS: time point 2 (t2) and time point 3 (t3)).** **p < 0.01 (t2 vs. t1); *p < 0.05 (t3 vs. t2).

Several of the skin physiology parameters worsened significantly during the observation period at the Enviromental Research Station Schneefernerhaus (UFS), e.g. skin hydration (decreased; p < 0.01), skin smoothness (increased; p < 0.01), skin roughness (increased; p < 0.05), skin scaliness (increased; p < 0.01) and pH-value (increased; p < 0.01). For details see Table 2. Histamine induced itch decreased significantly (Figure 1). The score of the RQLQ improved significantly (p < 0.05). Several lung parameters showed a slight, but statistically significant (p < 0.05) improvement (FEV1/VC, PEF, MEF 50, MMFEF 25/75), whereas the vital capacity (VC) decreased significantly (p < 0.05). Values of the measurement of fractional exhaled nitric oxide (FeNO) did not differ significantly at the different time points. In the serum a significant decrease (p < 0.01) of ECP was found at the beginning of the stay at the UFS versus the first assessment in Munich (t2 vs. t1) and again a significant increase for ECP (p < 0.01) at the last assessment in Munich versus the end of the stay at the UFS (t4 vs. t3). Parameters of blood count changed significantly. Data for IL-33 were too low to compare.

**Table 2:** Significant results of skin, nasal, conjunctival and lung parameters, questionnaires and blood parameters at the beginning (t1) in Munich, at time point 2 (t2) and 3 (t3) in the UFS and time point 4 (T4) in Munich in the different groups: data are means ± SD

**Parameter**	**t1 (pat.)**	**t1 (contr.)**	**t2 (pat.)**	**t2 (contr.)**	**t3 (pat.)**	**t3 (contr.)**	**t4 (pat.)**	**t4 (contr.)**	**Wilcoxon**	**ANOVA**
**Skin parameters**										
Str. corneum hydration (arb. units)	46±10	48±7	36±8	39±6	38±7	39±5	44±14	47±4	**t1/t2 **t3/t4	
PH	4.9±1.3	5.2±1.2	5.9±1.3	5.4±1.2	5.9±1.3	5.4±1.2	5.2±1.3	4.5±1.2	**t1/t2 **t3/t4	
Skin roughness (arb. units)	0.6±0.3	0.5±0.2	1.0±0.3	0.6±0.2	0.6±0.4	0.5±0.2	0.6±0.4	0.5±0.2	*t1/t2 **t3/t2	
Skin scaliness (arb. units)	0.3±0.2	0.3±0.2	0.7±0.3	0.7±0.4	0.8±0.3	0.6±0.3	0.6±0.3	0.5±0.2	**t1/t2 **t3/t4	
Skin smoothness (arb. units)	18.2±2.0	18.2±3.8	21.0±3.8	18.8.±4.4	22.3±4.3	18.5±4.3	19.0±2.9	15.1±0.5	**t1/t2 **t3/t4	↓AE t1/t2
TEWL (g/(hm^2^))	8.0±5.0	6.0±1.4	6.2±2.5	5.9±1.2	7.1±3.8	7.0±1.8	9.8±9.2	7.5±2.2		↑AE t3/t4
Blood flow (right) (arb. units)	109±23	96±16	n.d.	n.d.	121±49	97±19	108±15	98±16		↑AE t1/t3
Blood flow (left) (arb. units)	115±28	98±15	n.d.	n.d.	124±60	96±23	113±28	101±20		↑AE t1/t3 ↓AEt3/t4
Wheal diluted 1:10 (mm)	6.2±2.9	n.d.	5.7±2.3	n.d.	6.1±2.6	n.d.	4.9±1.6	n.d.		↑AR t1/t2
Wheal diluted 1:100 (mm)	2.9±1.1	n.d.	2.9±1.1	n.d.	3.6±1.2	n.d.	2.8±1.0	n.d.	*t2/t3	
Flare diluted 1:10 (mm)	31.3±10.1	n.d.	25.1±10.8	n.d.	23.4±8.5	n.d.	25.0±9.6	n.d	**t1/t2	
**Nasal and conjunctival parameters**										
Resistance (after prov.) (ml/s)	1.1±1.8	1.3±2.1	0.7±0.6	0.6±0.6	0.5±0.3	0.6±0.5	0.5±0.5	0.7±0.6		↑Asthma t2/t3
Conjunctival provocation (score) Allergen 1/100 (5min)	0.7±0.5	n.d.	0.4±0.5	n.d.	0.5±0.5	n.d.	0.6±0.5	n.d.		↑Asthma t1/t2 ↓Asthma t2/t3
**Lung parameters**										
FVC (l)	5.1±1.7	4.6±1.0	5.2±2.0	5.0±1.3	4.6±1.2	4.7±1.2	4.7±1.5	5.0±1.0	*t2/t3	
FEV1/VC (%)	78±15	88±12	80±17	89±8	85±11	90±7	81±14	89±9	*t1/t3	
PEF (l/s)	9.1±2.9	10.0±2.63	10.1±2.5	11.2±3.0	9.6±2.5	10.9±2.9	9.9±2.7	10.2±2.4	**t1/t2 **t2/t3 *t3/t4	
MEF50 (l/s)	4.26±1.7	5.35±2.0	4.76±2.0	5.84±2.1	4.85±1.9	5.60±1.8	4.67±1.5	5.5±1.4	*t1/t2	
MMEF 25/75 (l/s)	3.75±1.5	4.73±1.6	4.23±1.8	5.19±1.8	4.22±1.6	5.02±1.6	4.25±1.1	5.02±1.1	**t1/t2	
Resistance after 0.1% methacholine (kPA*s/l)	0.33±0.1	0.23±0.02	0.42±0.2	0.31±0.2	0.52±0.2	0.24±0.1	n.d.	n.d.	**t1/t2 **t1/t3	↓Asthma t1/t2 ↑AE t1/t2
FeNO (ppb)	41.6±34.6	17.6±7.9	32.0±24.9	12.8±10.2	38.2±34.3	20.0±15.4	33.2±26.5	16.2±9.3		
**Questionnaires**										
DIELH (score)	30±30	1±1	27±30	1±1	29±29	1±2	24±28	0±1		↑AE t1/t2
RQLQ (score)	5.6±0.9	6.6±0.3	5.6±1.0	6.8±0.2	5.8±0.9	6.8.±0.2	5.7±0.9	6.6±0.3	*t1/t3	↓Asthma t2/t3 ↑AE t2/t3
SF-36 (score)	2.2±0.4	2.0±0.4	2.2±0.4	2.0±0.5	2.2±0.4	2.0±0.5	2.4±0.2	2.2±0.2		↓AR t2/t3
**Blood parameters**										
Erythrocytes (10^6^/μl)	4.85±0.4	4.85±0.6	5.27±0.5	5.09±0.92	4.92±0.42	4.86±0.57	5.01±0.37	4.79±0.4	**t1/t2 **t2/t3	↓AR t2/t3
Hemoglobin (g/dl)	14.2±1.5	14.1±1.7	15.3±1.8	14.9±2.6	14.4±1.5	14.0±1.6	14.5±1.4	13.8±1.20	**t1/t2 *t2/t3*t3/t4	
Hematocrit (%)	42.9±3.7	42.3±4.5	46.6±4.6	44.3±6.7	43.4±3.6	42.6.0±4.0	44.8±3.2	43.0±2.90	**t1/t2 *t2/t3	↓AR t2/t3
Eosinophils (%)	4.0±3.1	1.9±1.3	4.3±2.9	2.0±1.2	4.9±3.1	3.1±1.8	4.2±3.1	2.3±1.1	**t1/t3	
Basophils (%)	0.5±0.2	0.4±0.2	0.4±0.2	0.3±0.2	0.5±0.2	0.5±0.2	0.5±0.2	0.3±0.20	**t1/t3	
ECP (μg/ml)	16.0±11.0	6.0±4.0	5.0±5.0	2.0±2.0	11.0±11.0	6.0±6.0	14.0±9.0	5.0±3.0	**t1/t2 **t2/t3 **t3/t4	
IL-33 (ng/ml)	2.96±4.04	4.16±5.98	2.81±4.1	4.0±5.92	2.72±3.91	3.72±5.97	1.85±2.45	5.16±.6.94	*t1/t2	↑Asthma t3/t4

*Abbreviations*: *AE* atopic eczema, *AR* allergic rhinitis, *arb. units* arbitrary units, *contr.* controls, *DIELH* Deutsches Instrument zur Erfassung der Lebensqualität bei Hauterkrankungen, *ECP* eosinophil cationic protein, *FeNO* fractional exhaled nitric oxide, *FEV1/VC* forced expiratory volume in one second/volume capacity, *FVC* forced volume capacity, *MEF50* maximum expiratory flow at 50% of vital capacity, *MMEF* 25/75 maximal mid-expiratory flow between 25% and 75% of vital capacity, *n.d.* not done, *pat.* patients, *PEF* peak expiratory flow, *ppb* parts per billion, *RQLQ* Rhinitis Quality of Life Questionnaire, *prov.* provocation, *Str.* stratum, *TEWL* transepidermal water loss, *p < 0.05, **p < 0.01.

This study shows, that a 5-day-sojourn at the Environmental Research Station Schneefernerhaus (UFS Zugspitze) at an altitude of 2650 m alt. exerts different effects on atopic diseases. A limitation of the study is the small number of participants.

Despite benefits of climate therapy in patients with atopic eczema during treatment in specialized in-patient facilities in the alpine mountain climate of Bavaria [11,12] or in mountain altitude conditions like Davos [1] we didn’t find a clinical amelioration of atopic eczema skin lesions using the SCORAD. Moreover some skin physiology parameters worsened (e.g. stratum corneum hydration, pH, skin roughness). On the one hand this might be due to the fact that topical glucocorticosteroids were withdrawn 1 week before time point 1, on the other hand side low air humidity at this altitude might have negative influences on the skin. Only histamine-induced itching showed a significant improvement during the observation period at the UFS. It is known that in the Swiss mountain area of Davos itch intensity was found to be correlated with some meteorological variables, especially air temperature [13].

An improvement of several lung parameters was observed, only the forced vital capacity decreased. There are several studies showing that FVC decreases with increasing altitude. It is assumed that this effect is related to pulmonary interstitial changes due to pulmonary artery enlargement and interstitial edema [14]. Improvement of other lung function disturbances had also been reported in previous studies with children and adolescents in in-patient rehabilitation programs [15-18] in moderately and high mountain climate. This may be due to a reduced allergen exposure in the altitude. House dust mites allergens haven’t been measured during this study, but it is known that mites can not survive in alpine mountain climate above 1500 m. Furthermore it could be shown that in general the amount of pollen (birch, grass) at the UFS is considerably lower than the amount in Munich (unpublished observations, Buters J et al.). Decrease of exhaled NO as a parameter of lung inflammation was also seen in asthmatic patients under mountain climate therapy [6], but not in our study. This may be due to the short time period at the UFS.

In the AURA (Allergien und Umweltkrankheiten in der Rehabilitation) study we found a significant increase of the SF-36 questionnaire confirming the benefit of the therapy in Pfronten [11], but in our study at the UFS there were no significant differences at the different time points. Also the skin specific questionnaires emphazising the pruritus (The Eppendorf Itch Questionnaire) and the quality of life (DIELH) showed a significant drop of the total score in the DIELH in the AURA study [11], but in our study these scores did not differ significantly. This discrepancy may be due to additional therapies (e.g. optimized therapy with glucocorticosteroids and systemic drugs) and trainings (e.g. special trainings for patients with asthma and atopic eczema) offered in a clinical department.

As expected exposure to moderate altitude had significant effects on red blood cells [19]. We could show an increase of eosinophils during the stay at the UFS. This is in contrast to other studies during hospital treatment of atopic eczema in the mountain climate [20] and the North Sea climate with decreased eosinophils [21].

Elevated ECP levels are regarded as markers of inflammation in asthma and atopic eczema [22]. A decrease of ECP had been shown in the mountain climate of Davos [23]. In our study, in patients with atopic diseases ECP decreased significantly from time point t1 to time point t2, but increased again at time point t3. This may reflect the aggravation of the skin parameters. It could be shown that circulating Il-16 levels are correlated with the SCORAD in adult patients with atopic eczema [24] and decreased significantly in these patients after successful treatment [25]. According to this, we did not find a decrease in our patients with atopic diseases.

The 5 day observation period in the mountain climate of the Zugspitze in Bavaria showed favourable results only for a limited number of parameters. Especially patients with asthma had a benefit from this stay, whereas skin physiology parameters worsened. This might be due to the short duration of the sojourn and typical environmental factors at this altitude. This pilot study suggests that it would be of interest to assess the skin parameters and characteristics of atopic eczema over a longer period of observations.

## Competing interests

The authors declare that they have no competing interests.

## Authors’ contributions

BE, RF, UD, HB, RH, JR have made substantial contributions to the conception and design of the study. RH, JHM, FP, RF, MS, JL, ML, VA, JL, CE, JB have made substantial contributions to the acquisition of the data. MK, RH, JL have made substantial contributions to the analysis of the data. BE and JR have been involved in drafting the manuscript. All other authors revised it critically, gave final approval and agreed to be accountable for all aspects of the work.
